# Genetic distance in the whole-genome perspective on *Listeria monocytogenes* strains F2-382 and NIHS-28 that show similar subtyping results

**DOI:** 10.1186/s12866-014-0309-0

**Published:** 2014-12-10

**Authors:** Daisuke Kyoui, Hajime Takahashi, Satoko Miya, Takashi Kuda, Shizunobu Igimi, Bon Kimura

**Affiliations:** Department of Food Science and Technology, Faculty of Marine Science, Tokyo University of Marine Science and Technology, 4-5-7 Konan, Minato, Tokyo, 108-8477 Japan; Division of Biomedical Food Research, National Institute of Health Science, 1-18-1 Kamiyoga, Setagaya-ku, Tokyo, 158-8501 Japan

**Keywords:** *Listeria monocytogenes*, MLVA, MVLST, Next-generation sequencing, Whole-genome shotgun sequence

## Abstract

**Background:**

Genome subtyping approaches could provide useful epidemiological information regarding food pathogens. However, the full genomic diversity of strains that show similar subtyping results has not yet been completely explored. Most subtyping methods are based on the differences of only a portion of the genome. We investigated two draft genome sequences of *Listeria monocytogenes* strain F2-382 and NIHS-28, which have been identified as closely related strains by subtyping (identical multi-virulence-locus sequence typing and multiple-locus variable number tandem repeat analysis sequence types and very similar pulsed-field gel electrophoresis patterns), despite their different sources.

**Results:**

Two closely related strains were compared by genome structure analysis, recombination analysis, and single nucleotide polymorphism (SNP) analysis. Both genome structure analysis and recombination analysis showed that these two strains are more closely related than other strains, from a whole-genome perspective. However, the analysis of SNPs indicated that the two strains differ at the single nucleotide level.

**Conclusion:**

We show the relationship between the results of genome subtyping and whole-genome sequencing. It appears that the relationships among strains indicated by genome subtyping methods are in accord with the relationships indicated by whole-genome analysis. However, our results also indicate that the genetic distance between the closely related strains is greater than that between clonal strains. Our results demonstrate that subtyping methods using a part of the genome are reliable in assessing the genetic distance of the strains. Furthermore, the genetic differences in the same subtype strains may provide useful information to distinguish the bacterial strains.

**Electronic supplementary material:**

The online version of this article (doi:10.1186/s12866-014-0309-0) contains supplementary material, which is available to authorized users.

## Background

*Listeria monocytogenes* is a rod-shaped, gram-positive, and non-sporulating foodborne infectious pathogen that can cause serious diseases such as septicemia and meningitis, particularly in high-risk groups (e.g., pregnant woman, neonates, and immunocompromised individuals) with a high mortality rate of 20%–30% [1]. In particular, ready-to-eat (RTE) foods, which do not require heat cooking, are a main source of foodborne listeriosis cases [2–4]. Molecular subtyping approaches allow us to evaluate the similarity of strains isolated from geographically or temporally different sources with high accuracy and reproducibility for epidemiological studies or trace-back surveys [5]. Several molecular subtyping methods such as PFGE, ribotyping, MLST including MVLST, and MLVA have been developed for subtyping of *L. monocytogenes* [6–10]*.* These methods have categorized the microorganism into 4 lineages and several clonal complexes (CCs). The subtyping data is highly consistent with pathogenicity and other characteristics, and provides useful information for epidemiologic or phylogenetic studies [11–13]. However, the whole-genome structure of strains that show similar subtyping results is unknown, because most subtyping methods focus on only a subset of loci such as endonuclease restriction sites or alleles.

Next-generation sequencing (NGS) technology has facilitated the analysis of bacterial genomes from a whole-genome perspective by generating relatively high data output in recent years. Using NGS, some studies have already reported the relationships between molecular subtyping results and whole-genome sequences for *Salmonella enterica* and *Escherichia coli*, and presented useful information on phylogeny and virulence [14–18]. *L. monocytogenes* has also been investigated at the whole-genome level to reveal its pathogenicity and evolutionary history [19–21]. However, the relationship between the results of genome subtyping and whole-genome sequencing in *L. monocytogenes* has not yet been fully explored.

We compared two draft genome sequences of *L. monocytogenes* to reveal the relationship between subtyping results and whole-genome diversity. Strains F2-382 and NIHS-28 were isolated from epidemic patients in the United States and Japan, respectively. These strains were not considered to be clonal because the patients had not engaged in travel around the time of onset. However, these strains showed identical MVLST and MLVA sequence types and strikingly similar PFGE patterns [22]. Thus, they are regarded to be extremely closely related strains, regardless of their source. In the present study, their draft sequences were compared to determine the extent of the differences between their genomes. In addition, published whole-genome sequences from 22 strains were compared to evaluate the genetic distance.

## Results

### General properties of the draft genome sequence

Using the shotgun sequencing method, a total of 46,603,422 bp and 107,127 reads with an average read length of 435.0 bp were obtained for the F2-382 strain. A total of 64,444,465 bp with 132,436 reads and an average read length of 486.6 bp were obtained for strain NIHS-28. Next, 99.58% of the total bp of the F2-382 strain and 99.71% of the total bp of the NIHS-28 strain were aligned in the *de novo* assembly. In total, 56 contigs were obtained from the *de novo* assembly of the F2-382 strain, with a total length of 2,911,674 bp, an N50-contig of 86,218 bp, and a GC content of 37.9%. The alignment depths were distributed from 1 to 140, with a peak depth of 13. For strain NIHS-28, 35 contigs were obtained with a total length of 2,908,138 bp, an N50-contig of 120,766 bp, and a GC content of 37.9%. The alignment depths were distributed from 1 to 180 with a peak depth of 19.

### Large-scale differences in genome structure

**Figure 1:**
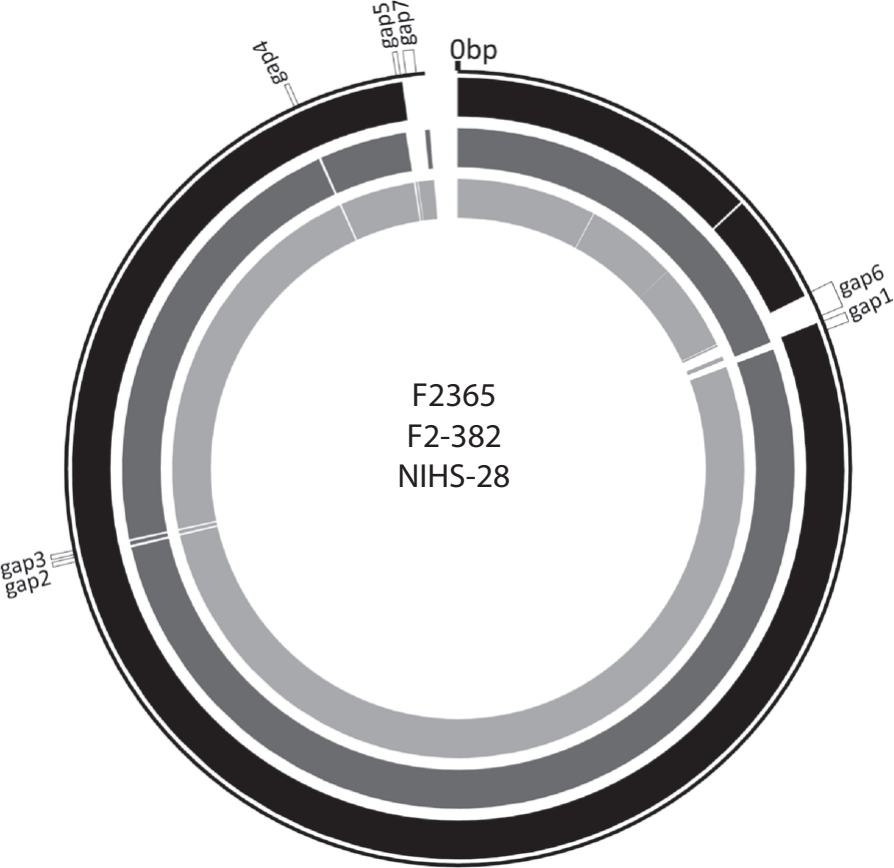
**Alignment of genome sequences of strains F2-382, NIHS-28, and F2365.** The seven frames on the outer ring show gap regions larger than 5 kbp. Each lane shows the sequence of one strain; F2365 (black); F2-382 (dark gray); NIHS-28 (light gray).

The alignment analysis constructed using MAUVE software for strains F2-382, NIHS-28, and F2365 showed that their overall genomic structures are similar (Figure 1). In addition, all of the contigs were aligned to the reference sequence, indicating that the sequenced strains did not possess plasmids. Five gap loci were identified in strains F2-382 and NIHS-28 when aligned to strain F2365 (Figure 1; gaps 1–5). However, between strains F2-382 and NIHS-28, only one locus for each sequence was identified as a gap (Figure 1; gap 6–7). Thus, strains F2-382 and NIHS-28 exhibit more similarity to each other than to F2365.

Each gap region between strains F2-382 and NIHS-28 contained several open reading frames (ORFs), which have been inserted by phage infection (Figure 1; gaps 6–7, Additional file 1 and Additional file 2). These gap regions were assumed to be derived from the individual phages, because they showed partial similarity in the alignment details for the region (Additional file 3).

### Recombination analysis

**Figure 2:**
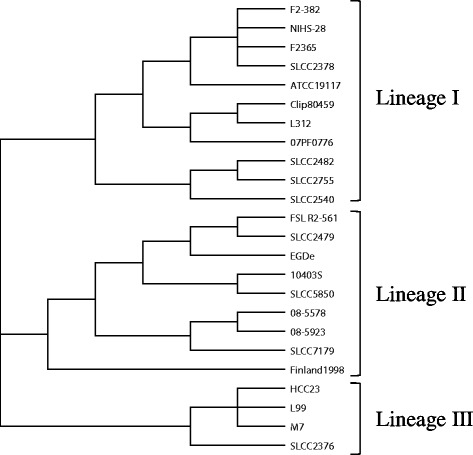
**The cluster diagram constructed by recombination analysis using a wide range of genome sequences.** Three independent datasets yielded identical clustering results.

All 24 strains were clustered into three major clades, which corresponded to previously described lineages (Figure 2, Table 1) [20,21,23–29]. Strains F2-382 and NIHS-28 were both clustered in the lineage I clade (Figure 2, Table 1). Lineage I was further divided into six clades. At the finest level of detail, strains F2-382 and NIHS-28 were clustered in the same clade as strains F2365 and SLCC2378 (Figure 2). Thus, strains F2-382 and NIHS-28 exhibit greater similarity to each other in their whole-genome sequence than to the other strains, consistent with the results of MVLST analysis and MLVA.

**Table 1:** **Isolates and genome subtyping results**

**Isolates**	**Genbank accession No.**	**Serotype**	**Lineage**	**MVLST** ^**a**^	**MLVA**	**Reference**
F2-382	BAZC01000001–01000056	4b	N/A	1-3-1-3-1-3	13-18-9	This study
NIHS-28	BAZD01000001–01000036	4b	N/A	1-3-1-3-1-3	13-18-9	This study
F2365	NC_002973	4b	I	1-3-1-3-1-3	14-18-9	Nelson et al. [25]
EGDe	NC_003210	1/2a	II	7-9-8-6-4-2	25-11-7	Glaser et al. [26]
HCC23	NC_011660	4a	III	4-5-4-1-*-4	8-22-4	Steele et al. [27]
Clip80459	NC_012488	4b	I	2-4-2-5-2-3	15-20-5	Hain et al. [20]
08-5578	NC_013766	1/2a	II	7-8-7-6-3-1	34-22-7	Gilmour et al. [28]
08-5923	NC_013768	1/2a	II	7-8-7-6-3-1	34-22-7	Gilmour et al. [28]
L99	NC_017529	4a	III	4-5-4-1-*-4	8-22-4	Hain et al. [20]
M7	NC_017537	4a	N/A	5-5-4-1-*-4	8-22-4	Chen et al. [23]
10403S	NC_017544	1/2a	N/A	8-12-6-6-3-2	20-11-7	den Bakker et al. [29]
FSL R2-561	NC_017546	1/2c	N/A	7-9-8-6-4-2	21-11-7	den Bakker et al. [29]
Finland1998	NC_017547	1/2a	N/A	7-8-7-6-3-2	25-14-7	den Bakker et al. [29]
07PF0776	NC_017728	4b	N/A	2-4-2-5-2-3	14-18-9	McMullen et al. [24]
ATCC19117	NC_018584	4a	I	2-1-3-3-1-3	17-13-6	Kuenne et al. [21]
SLCC2378	NC_018585	4e	I	1-3-1-3-1-3	17-18-9	Kuenne et al. [21]
SLCC2540	NC_018586	3b	I	3-7-2-4-2-3	10-11-5	Kuenne et al. [21]
SLCC2755	NC_018587	1/2b	I	2-6-3-3-2-3	15-16-5	Kuenne et al. [21]
SLCC2479	NC_018589	3c	II	7-9-8-6-4-2	21-11-7	Kuenne et al. [21]
SLCC2376	NC_018590	4c	III	6-2-5-2-*-4	9-17-4	Kuenne et al. [21]
SLCC2482	NC_018591	7	I	2-6-3-3-2-3	15-16-6	Kuenne et al. [21]
SLCC5850	NC_018592	1/2a	II	*-10-6-6-3-2	19-11-7	Kuenne et al. [21]
SLCC7179	NC_018593	3a	II	7-11-6-6-3-2	10-12-9	Kuenne et al. [21]
L312	NC_018642	4b	I	2-4-2-5-2-3	15-15-5	Kuenne et al. [21]

^a^Asterisks (*) indicate that the genome region corresponding to the allele was not found.

### A comparison of the clustering results between methods

**Figure 3:**
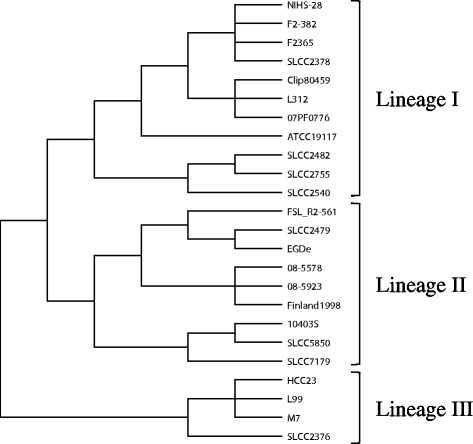
**The cluster diagram constructed by MVLST analysis using six virulence gene alleles.**

**Figure 4:**
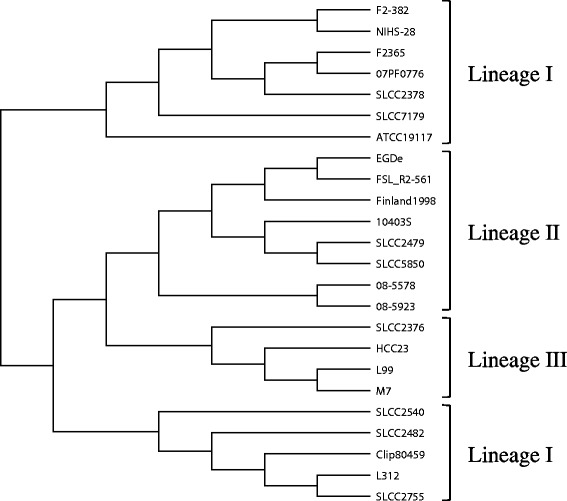
**The cluster diagram constructed by MLVA using three tandem repeat regions.**

Recombination analysis of a set of large-genome sequences showed some differences in the relationships between lineages, compared to other subtyping methods (Table 1; Figures 2, 3 and 4). In the recombination analysis, the three lineages clustered independently. In contrast, MVLST subtyping showed that lineages I and II are more closely related to each other than to lineage III (Figure 3). MLVA subtyping showed that lineage I could be separated into two major clusters, and that lineage II and III comprised a large cluster that did not include lineage I. However, the relationships among the strains within the lineage cluster were similar in all methods.

The results of MVLST analysis and MLVA of strains F2-382 and NIHS-28 were similar to those of strains F2365 and SLCC2378, which were identical to the results of recombination analysis. These four strains showed the same sequence type in MVLST analysis. MLVA results showed that strains F2-382 and NIHS-28 had the same MLVA type, and were distinct from other strains. In addition, strains F2365, SLCC2378, and 07PF0776 showed MLVA types similar to those exhibited by strains F2-382 and NIHS-28, and comprised a parent cluster.

### Single nucleotide polymorphism analysis of the four most similar strains

**Figure 5:**
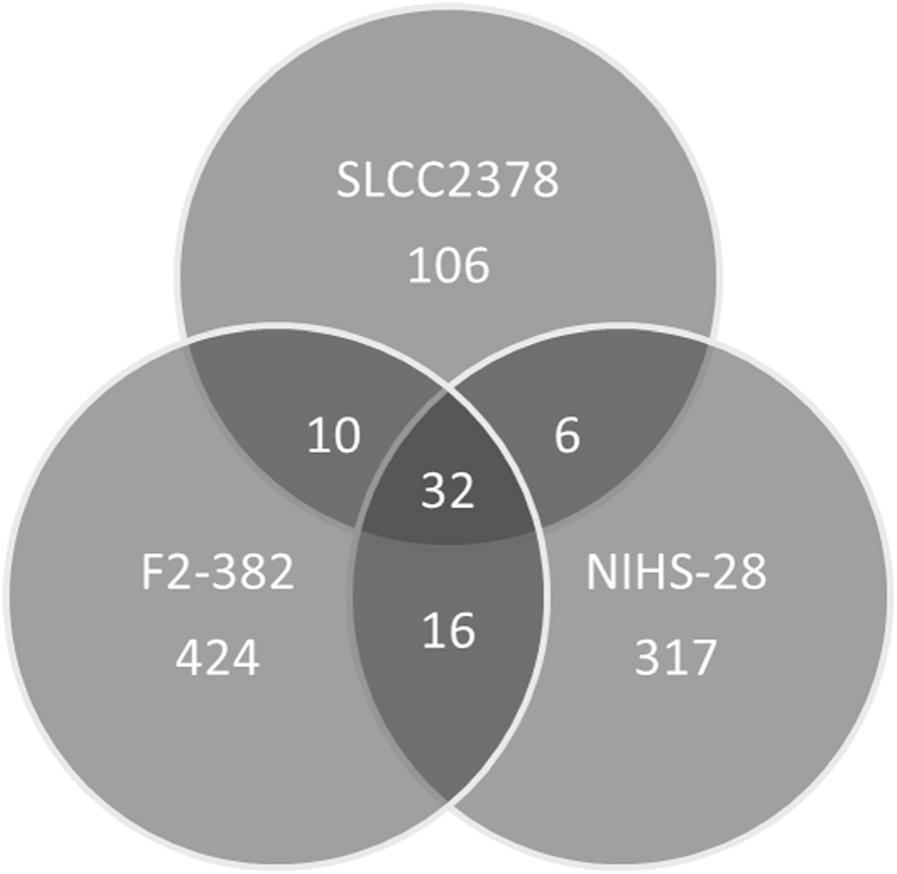
**The number of SNPs in each strain compared to strain F2365.** The numbers under each strain name indicate the number of SNPs in the strain. The numbers in the overlap region indicate the number of SNPs with the same polymorphism at the same locus between strains.

Strains F2-382, NIHS-28, F2365, and SLCC2378, which were clustered into the smallest clade in recombination analysis, were evaluated by single nucleotide polymorphism (SNP) analysis. Strains F2-382 and NIHS-28 were barely affected by the subculture, because they had been stored at the freezing temperature. Using strain F2365 as a reference, 424 SNPs were identified in strain F2-382, 317 SNPs in NIHS-28, and 106 SNPs in SLCC2378 (Figure 5). However, when comparing strains F2-382 and NIHS-28, only 48 SNPs were found at the same loci with the same polymorphism. Thus, most of the SNPs were unique to one of the two strains. The numbers of unique SNPs in strains F2-382 and NIHS-28 were 366 and 263, respectively, in contrast to 58 in the SLCC2378 strain. Thus, strains F2-382 and NIHS-28 have unique sequences, whereas strains F2365 and SLCC2378 have very similar sequences.

## Discussion

### Differences in genome structure

We examined the differences between the draft genome sequences of strains showing similar genome subtyping results by using NGS technology. The backbone of the genome (e.g., GC content, number of rRNAs or tRNAs) is known to be similar among the different strains of *L. monocytogenes* regardless of the serotype and lineage, with the exception of a partial mutation by gene deletion and/or transfer [19,29]. Strains F2-382 and NIHS-28 also showed more similar genomic structure in the set of large genome sequences based on alignment with strain F2365, compared to the other strains. Furthermore, fewer gaps were observed between strains F2-382 and NIHS-28 compared to strain F2365. Thus, it appears that these two strains, which were shown to be closely related by genome subtyping analysis, are more similar to each other than they are to other strains in the genomic structure that is formed by insertion and deletion, not just in the genomic backbone of GC content.

Both F2-382 and NIHS-28 had only a single gap in their genomes (Figure 1; gap 6–7) when we set the minimum gap size as 5 kbp. The smaller gaps could be unreadable regions in the shotgun sequencing or could be generated by misassembly. Annotation of the region containing the gaps indicated that both gaps were derived from a phage infection (Additional file 1 and Additional file 2). Phage infection is a primary type of genome rearrangement that can adapt to a wide range of environments [20,21,25,30]. Interestingly, both prophage regions encode a gene related to sugar phosphorylation (Additional file 1 and Additional file 2; F2382_00310, LmNIHS28_02116). Several paralogs of this type of gene are known to be present in the genome, and play a role in energy production in host cells in the absence of sugar [20,31]. We can hypothesize that the genes encoded in the prophage region are a result of adaptation, as both F2-382 and NIHS-28 were isolated from epidemic patients.

### Genetic distance between strains F2-382 and NIHS-28

To compare genetic distances in the whole genome, Deloger et al. established a maximal unique matches index (MUMi) [32]. However, we were not able to calculate this index because the two draft whole-genome sequences obtained in this study were not of sufficient length for an accurate calculation. Therefore, we compared the strains in the aligned region by establishing three large datasets. Large dataset recombination analysis showed that the strains formed clusters corresponding to lineages, as described in a previous study (Figure 2, Table 1) [20,21,23–29]. It appears that the lineage perspective that is reflected by partial genome analysis is consistent with the relationships indicated by comparison of whole-genome sequences. This hypothesis has been supported by Kuenne et al., who reported that phylogenetic analysis using 2,018 gene sequences showed high correspondence with the lineages [21]. Furthermore, the present study shows that the set of large genome sequences, including non-functional regions, are associated with lineages.

Recombination analysis indicated that both strains F2-382 and NIHS-28 belong to the lineage I cluster. Lineage I can be classified into at least five CCs [12]. In the present study, the strains that clustered in lineage I formed the six smallest clades (Figure 2). Strains F2-382 and NIHS-28 were both identified as belonging to the smallest clade, indicating that they are more closely related than the other strains.

Strains F2365 and SLCC2378 were included in the same clade with strains F2-382 and NIHS-28 (Figure 2). Kuenne et al. reported that these two strains have extremely similar genome sequences [21]. We used SNP analysis to compare the four strains to determine genomic similarity at the single nucleotide level (Figure 5). One limitation of analysis using NGS technology is false-positive SNPs caused by sequencing errors. To avoid this problem, we excluded SNPs with quality scores of <40, ensuring an accuracy of >99.99%. Thus, since <1000 SNPs were detected, the number of false-positives should be <1. Furthermore, to avoid false-positive SNPs caused by homopolymers, which is particularly problematic in 454 sequencing, we detected only SNPs with depths of >10 in the assembly result. We also ensured that all sequence reads showed identical nucleotides at a given position. For SNP detection, strain F2365 was used as a reference for comparison with the other three strains. In strains F2-382 and NIHS-28, the majority of the SNPs detected were unique to each strain (F2-382, 366; NIHS-28, 263). However, only 10%–15% of the SNPs (F2-382, 48/424; NIHS-28, 48/317) involved identical mutations at the same locus. In contrast, only 58 SNPs were unique to strain SLCC2378. Gilmour et al. compared complete genome sequences of strains isolated from the same outbreak, and reported only 36 SNPs [28]. Based on the above results, it appears that the genome sequences of strain F2-382 and NIHS-28 are very similar, but less similar than those of clonal strains.

### Relationship between genome subtyping results and whole-genome sequencing

The recombination analysis result in the present study generated from an accumulation of point mutations. Accordingly, this result can be considered to indicate the vertical relationship among the strains. When the recombination analysis result was compared to MVLST analysis and MLVA, different relationships were observed for each subtyping method between clusters corresponding to lineages. The three lineages were shown to be independent by large genome sequence recombination analysis. In contrast, MVLST indicated that lineages I and II were more closely related to each other than to lineage III. We presume that this is because MVLST is based only on virulence genes [33]. Thus, the lineage III strains, which were rarely associated with human listeriosis cases, were located distantly from lineage I and II strains. The relationships between lineages indicated by MLVA also differed from the results of other analyses. It is known that the number of tandem repeats can be altered several times in a short period of time in *E. coli* O157:H7 and *Vibrio parahaemolyticus* [34,35]. Based on this knowledge, we assumed that the characteristics of the tandem repeat region biased the results. While the relationships among the lineages are different, as previously mentioned, the relationships among the strains under the lineage cluster were almost identical in all analyses. Thus, genome subtyping methods are valid for the identification of strains because the alleles require adequate time for mutation. In addition, we concluded that the results of genome subtyping are correlated with the phylogenetic relationships between strains when considering a limited group such as a lineage.

### Accuracy of genome subtyping methods

Our results suggest that strains that show similar subtyping results have similar whole-genome sequences, according to the analysis of DNA sequence, which is the origin of the genome subtyping methods. In a limited group of strains, the relationships indicated by the genome subtyping methods were correlated with the results of whole-genome sequencing. These results support the reliability of the phylogenetic relationships that were inferred using genome subtyping methods in previous studies. However, our observations also demonstrate that a more accurate classification is possible by single nucleotide analysis within the whole-genome perspective.

Strains F2-382 and NIHS-28, which showed similar subtyping results, were isolated from the United States and Japan, respectively. Listeriosis is rare in Japan, and only one foodborne case has been reported [36,37]. In contrast, cases have been sporadically reported in the United States and in European nations [38,39]. However, foodborne illnesses are no longer geographically limited since the globalization of food, including RTE foods, has been promoted worldwide [40–42]. Therefore, it is necessary to evaluate the virulence or to trace the origin of *L. monocytogenes* strains in foods [39,41]. The present study demonstrates that the genome typing results can be considered as genetic distances regardless of their geographically or temporally independent sources. This observation may improve the reliability of subtyping results in surveys.

The identity of individual strains from a whole-genome perspective is suggested by the present results. Genome-wide SNP analysis demonstrated that strains F2-382 and NIHS-28, which have been shown to be very similar by a previous subtyping method [22], are not clonal strains. The development of novel DNA sequencing technologies, including NGS, allows the analysis of microorganisms, including *L. monocytogenes,* from a whole-genome perspective [14–21]. We anticipate that the observations reported here will be useful as a reference for future studies that require validation of strain identity.

## Conclusions

We revealed the genome differences between two similar subtype strains, F2-382 and NIHS-28, from a whole-genome perspective, and estimated the concordance of the whole-genome sequence and genome subtyping results. Strains that show similar subtypes were shown to also have similar sequences at the whole-genome scale. We observed that the relationships among strains indicated by genome subtyping methods are consistent with the relationships indicated by whole-genome analysis. However, we also revealed that the genetic distance between closely related strains is greater than that between clonal strains, from a single nucleotide perspective. These findings could facilitate improvement of the reliability of genome subtyping results, in that they may be valuable references for estimating genetic distances by using subtyping methods. We anticipate that our findings will be useful for evaluating the identity of the strains.

## Methods

### Bacterial isolates

*L. monocytogenes* strain F2-382 was isolated from a patient in the United States and was kindly provided by Dr. Martin Wiedmann (Cornell University, Ithaca, NY). Strain NIHS-28 was isolated from a patient in Japan. Both strains were of serotype 4b. Ethical approval was not required, as the clinical isolates were collected as part of standard patient care. These strains showed identical MVLST and MLVA sequence types and only one difference in the PFGE band pattern [22]. However, they were considered as non-clonal strains, because the patients had not engaged in travel near the time of onset of listeriosis. After isolation, these strains had been stored at freezing temperature until pre-culture to extract DNA.

Whole-genome sequences were obtained for 22 additional strains from the GenBank/EMBL/DDBJ databases (http://www.insdc.org/) for comparison (Table 1) [20,21,23–29]. The strains contained in the reference showed nine serotypes (1/2a, 4a, 1/2b, 3b, 4b, 1/2c, 3c, 4c, and 7).

### Whole-genome shotgun sequence

Strains F2-382 and NIHS-28 were cultured overnight in brain-heart infusion broth (Eiken Chemical, Tochigi, Japan) at 37°C. Bacterial DNA was extracted using the phenol-chloroform extraction and ethanol precipitation methods [43,44]. For whole-genome shotgun sequencing, the GS Junior platform (Roche, Basel, Switzerland) was employed using a GS Junior Rapid Library Preparation Kit and a GS Junior emPCR Kit (Lib-L; Roche), according to the manufacturer’s protocol. The read sequences were used to construct a contig without a reference sequence by *de novo* assembly, using the GS *De Novo* Assembler (Roche). For this assembly, the program parameters were set to: seed step, 12; seed length, 16; seed count, 1; minimum overlap, 10; and minimum identity, 90.

### Genome structural analysis

To construct the scaffolds from the contigs, the software MAUVE v2.3.1 was employed, with the move contig script using the default settings [45]. Strain F2365 was chosen as a reference because it was aligned with the largest number of contigs in the 22 reference strains. The aligned contigs were constructed into a scaffold by joining with an N of 100 bp between each contig.

### Annotation

ORFs were extracted from each contig by using the Glimmer v3.02 software [46]. The ORFs were imported into the Genome Traveler software (In Silico Biology, Kanagawa, Japan) for annotation. The microbial database included in the software was used in combination with the BLAST algorithm for annotation. ORFs that showed less than 90% identity during the annotation process were excluded from subsequent analyses; this threshold indicated that the gene coding region had low reliability.

### Recombination analysis

Recombination analysis was conducted according to Cao et al.’s method [16]. To identify the core local collinear blocks (LCBs), which are the sequence segments common to all strains, the two scaffold sequences from F2-382 and NIHS-28 were aligned with the complete whole-genome sequences of the 22 reference strains with the MAUVE software, using the progressiveMauve script [47]. Using this alignment, 258 core LCBs were identified. Three independent data sets were constructed by randomly extracting approximately 500-kbp sequences from core LCBs, and evaluated by recombination analysis using ClonalFrame software v1.1 [48]. For this analysis, the parameters were set to 25,000 rounds of burn within the 50,000 generations and 100 thinning intervals. To rule out any bias among the three independent data sets, the results were evaluated using Gelman-Rubin statistics.

### *In silico* MVLST and MLVA

The genome regions for the targets of MVLST and MLVA were extracted for cluster analysis from the 24 *L. monocytogenes* sequences. The target loci for MVLST analysis were chosen according to Zhang et al.’s method [33]. The loci for MLVA were chosen according to Miya et al.’s method [22]. Clustering analysis was conducted using BioNumerics software v. 4.0 (Applied Maths, Sint-Martens-Latem, Belgium) by using the categorical coefficient and UPGMA method.

### Single nucleotide polymorphisms

SNPs were identified for four strains (F2-382, NIHS-28, F2365, and SLCC2378) that clustered in a single group in recombination analysis. MUMmer software v3.23 was employed to identify SNPs by alignment with the reference sequence, F2365 [49]. To avoid false-positives caused by sequencing errors and the problematic 454 sequencing-related homopolymers for strains F2-382 and NIHS-28, the polymorphisms that showed a quality score of <40 or a depth of <10 in the *de novo* assembly results were excluded from the SNP results.

### Availability of supporting data

The draft genome sequences of strains F2-382 and NIHS-28 were deposited in the Genbank/EMBL/DDBJ database with the accession numbers BAZC01000001–BAZC01000056 and BAZD01000001–BAZD01000035, respectively.

## Additional files

Additional file 1:
**F2-382 CDSs in the gap region.**
Additional file 2:
**NIHS-28 CDSs in the gap region.**
Additional file 3:
**The alignment result of gap 6 of strain F2-382 and gap 7 of strain NIHS-28.**

## Abbreviations

PFGE: Pulsed-field gel electrophoresis
MLST: Multi locus sequence typing
MVLST: Multi-virulence-locus sequencing typing
MLVA: Multi-locus variable number of tandem repeat analysis
ORF: Open reading frame
SNP: Single nucleotide polymorphism
NGS: Next-generation sequencing
LCB: Local collinear block
