# Supplementary material for: Genetic distance in the whole-genome perspective on Listeria monocytogenes strains F2-382 and NIHS-28 that show similar subtyping results
Source: BMC Microbiol. 2014 Dec 10;14:309. doi: 10.1186/s12866-014-0309-0 (PMC4269915; doi:10.1186/s12866-014-0309-0)
Supplement: Additional file 1: — F2-382 CDSs in the gap region. [file 12866_2014_309_MOESM1_ESM.pdf]

Additional file 1 F2-382 CDSs in the gap region

Contig	Position	Locus tag	Product	Note
Lm_F2-382_contig00002	149844..150377	F2382_00310	6-phospho-beta-glucosidase	COG2723; Beta-glucosidase/6-phospho-beta-glucosidase/beta-
	150383..150838	F2382_00311	hypothetical protein	
	151266..152192	F2382_00312	N-acetylmuramoyl-L-alanine amidase domain containing protein	Peptidase family U32; cl03113
	153086..153538	F2382_00313	hypothetical protein	
	154051..155118	F2382_00314	bacteriophage scaffolding GP20 family	
	156141..157175	F2382_00315	tail tape measure protein gp18	
	158002..160044	F2382_00316	putative tape-measure protein	
	160062..163367	F2382_00317	putative tape-measure protein	
	163378..163983	F2382_00318	gp15 protein	Bacteriophage Gp15 protein;
	163989..164411	F2382_00319	hypothetical protein	Phage protein Gp14; pfam10666
	164726..165163	F2382_00320	putative major tail shaft protein	
	165166..165573	F2382_00321	gp11protein	
	165911..166273	F2382_00322	hypothetical protein	Minor capsid protein; pfam10665
	166273..166677	F2382_00323	hypothetical protein	
	166828..167727	F2382_00324	conserved protein of unknown function	Phage capsid family; pfam05065
	167751..168320	F2382_00325	minor structural protein Gp20	Phage minor structural protein GP20; pfam06810
	168399..169538	F2382_00326	putative 3-mercaptopyruvate	
	169544..171043	F2382_00327	phage portal protein, putative, A118 family	
	171047..172423	F2382_00328	pbsx family phage terminase large subunit	
	172929..173228	F2382_00329	phage protein	
	173519..173953	F2382_00330	gp66 protein	
	174265..174648	F2382_00331	gp59 protein	
	174652..175119	F2382_00332	gp58 protein	Protein of unknown function (DUF1064); pfam06356
	175211..175690	F2382_00333	single-stranded DNA-binding protein	Helix-destabilizingprotein
	175712..176215	F2382_00334	hypothetical protein	Protein of unknown function (DUF812); pfam05667
	176885..177172	F2382_00335	hypothetical protein	
	177404..177769	F2382_00336	conserved protein of unknown function	
	179446..180057	F2382_00337	hypothetical protein	
	181434..182348	F2382_00338	gp49 protein	
	183290..184816	F2382_00339	hypothetical protein	
	185433..185957	F2382_00340	gp43 protein	
	186079..186882	F2382_00341	hypothetical protein	
	186898..187275	F2382_00342	hypothetical protein	
	187838..188323	F2382_00343	gp33 protein	Predicted transcriptional regulators[Transcription]; COG1396 Helix-turn-helix XRE-family like proteins.Prokaryotic DNA binding proteins belonging to thexenobiotic response element family of transcriptionalregulators; cd00093 salt bridge
	188381..188710	F2382_00344	hypothetical protein	
	189179..189403	F2382_00345	gp15 protein	
	189418..190104	F2382_00346	putative lipoprotein gp32	
	190147..191373	F2382_00347	putative integrase/recombinase	Int/Topo IB signature motif
