# Supplementary material for: Genetic distance in the whole-genome perspective on Listeria monocytogenes strains F2-382 and NIHS-28 that show similar subtyping results
Source: BMC Microbiol. 2014 Dec 10;14:309. doi: 10.1186/s12866-014-0309-0 (PMC4269915; doi:10.1186/s12866-014-0309-0)
Supplement: Additional file 2: — NIHS-28 CDSs in the gap region. [file 12866_2014_309_MOESM2_ESM.pdf]

Additional file 2 NIHS-28 CDSs in the gap region

Contig	position	Locus tag	Product	Note
Lm_NIHS-28_contig00024	48..1481	LmNIHS28_02108	putative integrase	
	2689..3174	LmNIHS28_02109	gp33 protein	
	3922..4695	LmNIHS28_02110	phage protein	Phage antirepressor protein KilAC domain;pfam03374
	4817..5350	LmNIHS28_02111	gp43 protein	
	6077..7603	LmNIHS28_02112	hypothetical protein	
	8545..9459	LmNIHS28_02113	gp49 protein	
	10013..10825	LmNIHS28_02114	DNA adenine	
	11379..11939	LmNIHS28_02115	hypothetical protein	
	12103..12561	LmNIHS28_02116	hypothetical protein	
	13117..13515	LmNIHS28_02117	gp59 protein	
	13694..14176	LmNIHS28_02118	single-stranded DNA-binding protein	Single-stranded DNA-binding protein [DNAreplication, recombination, and repair]; COG0629<br>Helix-destabilizingprotein<br>dimer interface [polypeptide binding]<br>tetramer (dimer of dimers) interface [polypeptidebinding]
	14616..15020	LmNIHS28_02119	integrase	
	15390..15980	LmNIHS28_02120	putative Positive control factor	positive control sigma-like factor; Validated<br>DNA binding residues [nucleotide binding]
	16191..16469	LmNIHS28_02121	conserved protein of unknown function	
	17212..18552	LmNIHS28_02122	phage terminase, large subunit, PBSX family	Phage terminase large subunit; cl12054<br>Terminase-like family; pfam03237
	18567..20123	LmNIHS28_02123	hypothetical protein	
	20128..21171	LmNIHS28_02124	gp4 protein	Phage Mu protein F like protein; cl10072
	21267..21821	LmNIHS28_02125	phage protein	
	21844..22716	LmNIHS28_02126	putative phage major capsid protein	
	23221..23616	LmNIHS28_02127	hypothetical protein	
	24275..24982	LmNIHS28_02128	Ig-like virion protein	
	25032..25463	LmNIHS28_02129	hypothetical protein	
	25776..30575	LmNIHS28_02130	phage tail tape measure protein	
	30579..31403	LmNIHS28_02131	hypothetical protein	
	33451..34524	LmNIHS28_02132	gp20 protein	
	35037..35411	LmNIHS28_02133	gp23 protein	
	35450..35725	LmNIHS28_02134	holin	
	35941..36867	LmNIHS28_02135	N-acetylmuramoyl-L-alanine amidase domain containing protein	
	37219..37773	LmNIHS28_02136	aspartate-semialdehyde dehydrogenase	COG0136 Aspartate-semialdehyde dehydrogenase<br>aspartate-semialdehyde dehydrogenase; Provisional<br>Semialdehyde dehydrogenase, dimerization
