# Supplementary material for: Genetic distance in the whole-genome perspective on Listeria monocytogenes strains F2-382 and NIHS-28 that show similar subtyping results
Source: BMC Microbiol. 2014 Dec 10;14:309. doi: 10.1186/s12866-014-0309-0 (PMC4269915; doi:10.1186/s12866-014-0309-0)
Supplement: Additional file 3: — The alignment result of gap 6 of strain F2-382 and gap 7 of strain NIHS-28. [file 12866_2014_309_MOESM3_ESM.pdf]

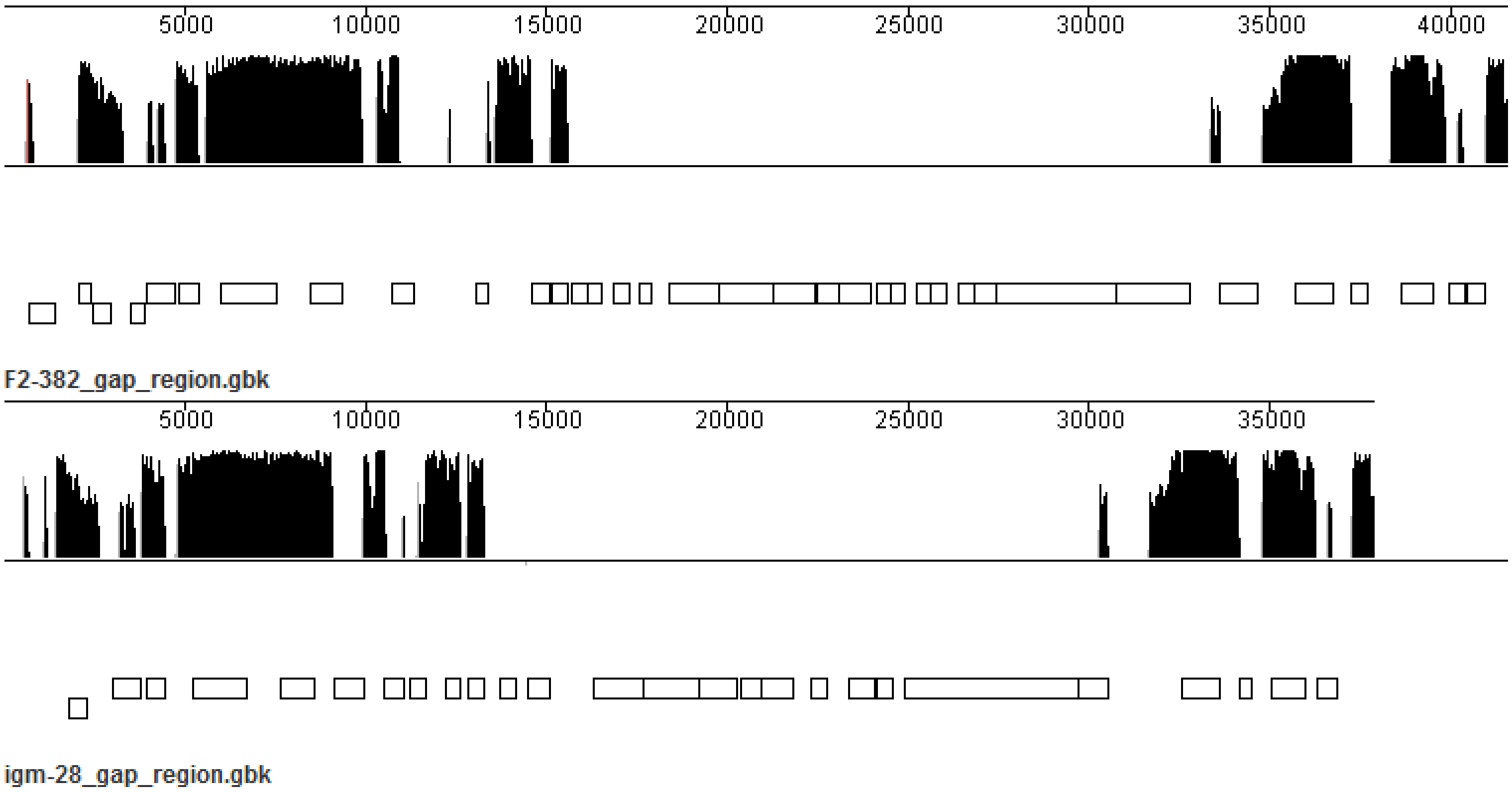

Additional file 3 The alignment result of gap 6 of strain F2-382 and gap 7 of strain NIHS-28. The upper lane shows gap6. And the lower lane shows gap 7. In the each lane, the peaks show the similarity between the gap regions. The squares in the bottom of lane show the ORFs.
